# The impacts of COVID-19 pandemic on service delivery and treatment outcomes in people living with HIV: a systematic review

**DOI:** 10.1186/s12981-022-00496-7

**Published:** 2023-01-06

**Authors:** SeyedAhmad SeyedAlinaghi, Pegah Mirzapour, Zahra Pashaei, Arian Afzalian, Marcarious M. Tantuoyir, Roghayeh Salmani, Seyed Farzad Maroufi, Parinaz Paranjkhoo, Seyede Parmis Maroufi, Hajar Badri, Sanaz Varshochi, Farzin Vahedi, Esmaeil Mehraeen, Omid Dadras

**Affiliations:** 1 Iranian Research Center for HIV/AIDS, Iranian Institute for Reduction of High Risk Behaviors, Tehran University of Medical Sciences, Tehran, Iran; 2 School of Medicine, Tehran University of Medical Sciences, Tehran, Iran; 3 Biomedical Engineering Unit, University of Ghana Medical Center (UGMC), Accra, Ghana; 4 Department of Midwifery, Khalkhal University of Medical Sciences, Khalkhal, Iran; 5 Turpanjian College of Health Sciences, American University of Armenia, 0019 Yerevan, Armenia; 6 Faculty of Medicine, Semnan University of Medical Sciences, Semnan, Iran; 7 Department of Health Information Technology, Khalkhal University of Medical Sciences, Khalkhal, 5681761351 Iran; 8 Department of Global Public Health and Primary Care, University of Bergen, Bergen, Norway

**Keywords:** COVID-19, Services, Treatment outcomes, PLHIV, SARS-CoV-2, HIV, AIDS

## Abstract

**Introduction:**

The COVID-19 epidemic and various control and mitigation measures to combat the widespread outbreak of the disease may affect other parts of health care systems. There is a concern that the COVID-19 pandemic could disrupt HIV services. Therefore, this study aimed to systematically evaluate the effect of the COVID-19 pandemic on service delivery and treatment outcomes in people with HIV.

**Methods:**

In this study, a systematic search was conducted using the keywords in the online databases including Scopus, PubMed, Web of Science, and Cochrane databases. The retrieved articles underwent a two-step title/abstract and full-text review process, and the eligible papers were selected and included in the qualitative synthesis.

**Result:**

We selected 16 studies out of 529 retrieved records that met the inclusion criteria for this review. Study populations of the selected studies were either HIV-positive patients or HIV clinics and healthcare providers. Most studies were focused on adhering to and obtaining medication and attending clinical appointments and their decrement during the pandemic. Other aspects of HIV care (alternative healthcare settings, viral suppression, psychological care, etc.) were discussed to a lesser extent by the included studies.

**Conclusion:**

Interruption in in-person visits and medical follow-up services, loss of adherence to treatment, and subsequent increase in mortality due to the COVID-19 pandemic complications in PLHIV have led to growing concerns. Other challenges were psychological disorders such as anxiety and depression, an increase in substance abuse, and a rise in experienced stigma and discrimination. However, the use of telemedicine in some countries helps to alleviate the situation to some extent and is recommended in similar settings in the future.

## Introduction

The first reports of severe acute respiratory syndrome coronavirus 2 (SARS-CoV-2) were released on December 31, 2019, from Wuhan, China, which was later declared a global public health emergency by the World Health Organization (WHO) in January 2020 [1, 2]. In March 2020, WHO declared coronavirus disease a global epidemic [3]. A recent report as of April 4, 2022, indicated that more than 492 million people have been infected with the virus, of which more than 6 million have died [4, 5].

Following the announcement of the WHO on the prevalence of coronavirus disease, countries have implemented various control and mitigation measures in their agenda to combat the spread of the virus [6]. Control measures included widespread testing, prompt contact tracing, quarantine, and mitigation measures, including hand hygiene, travel restrictions, school closures, social distancing, and total lockdowns [7]. Although these measures are considered necessary to control the epidemic, they can cause problems inadvertently affecting other public health programs; for example, many non-emergency outpatient clinical services were limited or suspended, many patients were assigned to receive remote treatment, drug supply was disrupted, budgets were reduced, virology laboratory priorities changed and most of their activities were directed toward COVID-19 diagnosis [8]. Most clinical staff were transferred to COVID-related activities, transportation was restricted, personal mobility was reduced; and generally, all parts of the health care system were affected [9, 10].

Chronic diseases generally account for about 60% of all deaths worldwide. HIV is one of the leading causes of death worldwide, with about 690,000 people dying each year [11, 12]. Global efforts to combat HIV have been hampered since the outbreak of the coronavirus (COVID-19), and global funding to respond to the disease has dwindled [13]. Some of the preventative measures associated with the COVID-19 epidemic such as physical distancing [14]; closure of social services; postponement of health appointments; and job loss can lead to social isolation, financial insecurity, and discontinuation of antiretroviral therapy (ART) medications [15]. The total number of people living with HIV by 2020 is estimated at 37.6 million worldwide, of which 1.3 million adults and 160,000 new infected children were identified in 2020 [16]. About 16% of people living with HIV (PLWH) were unaware of their HIV status, 27% were unable to receive ART, and 34% of those who received ART did not have viral suppression, and it is estimated that about 19% of HIV-infected patients were unable to receive a refill for antiretroviral drugs during the epidemic [17, 18]. Several modeling studies of the disruption of HIV programs by the COVID-19 epidemic have estimated that disruption of ART will have the greatest impact on HIV mortality [19, 20]. Therefore, our aim was to systematically evaluate the impact of COVID-19 pandemic on service delivery and treatment outcomes in people living with HIV.

## Materials and methods

### Study design and setting

This study is a systematic review of current evidence conducted in April 2022. The authors tried to investigate the impacts of the COVID-19 pandemic on service delivery and treatment outcomes in people living with HIV. This study adhered to the Preferred Reporting Items for Systematic Reviews and Meta-Analyses (PRISMA) to ensure the reliability and validity of the study results.

### Data sources

We extracted all the relevant papers and reports published in English through a systematic search in the online databases of PubMed (Medline), Web of Science, Scopus, Up-to-date, and Science Direct. Our search strategy employed multiple combinations of keywords, as follows:

A: [services] (Title/Abstract) OR [outcomes] (Title/Abstract) OR [follow-up] (Title/Abstract).

B: [HIV] (Title/Abstract) OR [AIDS] (Title/Abstract) OR [Human Immunodeficiency Virus] (Title/Abstract), [Acquired Immunodeficiency Syndrome] (Title/Abstract).

C: [Covid-19] (Title/Abstract) OR [SARS-COV-2] (Title/Abstract) OR [Corona virus] (Title/Abstract).

### Study selection

The selection of the studies was performed by assessing relevance based on the titles and abstracts by three independent investigators. The full texts were reviewed and assessed against the eligibility criteria. The English-written peer-reviewed original papers published were included.

The exclusion criteria:

- Non-human study papers including animal experiments, in vitro observations, and papers with no reference to the keywords of this review.
- Papers with unavailable full texts.
- Any duplicated and suspicious outcomes in databases.
- Review articles, editorial, commentaries, opinions, or any studies with no original data
- Ongoing projects (e.g., articles discussing the protocol of a future study)

### Data extraction

We used the data extraction forms to summarize the information related to the authors, year of publication, country, study population, age, gender, and reasons. Furthermore, in order to understand the HIV and non-HIV directed care challenges that PLWH experienced throughout the pandemic, we extracted compatible data from all eligible studies on the impact of COVID on HIV care services (whether HIV-specific or general services that PLWH require). This information was extracted by two independent investigators and utilized to construct the results section.

### Quality assessment

As mentioned, we used the PRISMA checklist to ensure the quality and reliability of the selected articles. Two independent researchers evaluated the quality of the articles and the risk of bias. And a third independent researcher addressed any raised discrepancies between them. The full text of the selected articles was read in its entirety and the main findings were extracted. Moreover, the Newcastle–Ottawa Scale (NOS) risk assessment tool was applied to determine the bias risk in the included studies. A total score of nine in three categories of selection, comparability, and exposure/ outcome with respective subtotal scores of four, two, and three (Table 1) is the measure of quality and bias risk assessment by NOS.

**Table 1:** Newcastle–Ottawa Scale (NOS) quality assessment of the study

First author	Selection (out of 4)	Comparability (out of 2)	Exposure/outcome (out of 3)	Total (out of 9)
Ballivian et al. [22]	***	**	**	7
Giacomelli et al. [43]	***	*	***	7
Hochstatter et al. [23]	****	**	***	9
Quiros-Roldan et al. [24]	****	**	***	9
Ridgway et al. [49]	***	**	***	8
Tamargo et al. [25]	***	*	***	7
Wagner et al. [26]	**	**	**	6
Ahmed et al. [27]	****	**	***	9
Campbell et al. [28]	***	**	***	8
Chilot et al. [29]	***	**	***	8
Karjadi et al. [30]	****	**	**	8
Nguyen et al. [31]	***	**	***	8
Rhodes et al. [33]	***	**	***	8
SieweFodjo et al. [21]	****	**	**	8
Yang et al. [32]	***	*	***	7
Qiao et al. [42]	***	**	***	8

The [*] indicates number(s) for each study regarding each column.

## Results

**Fig. 1:**
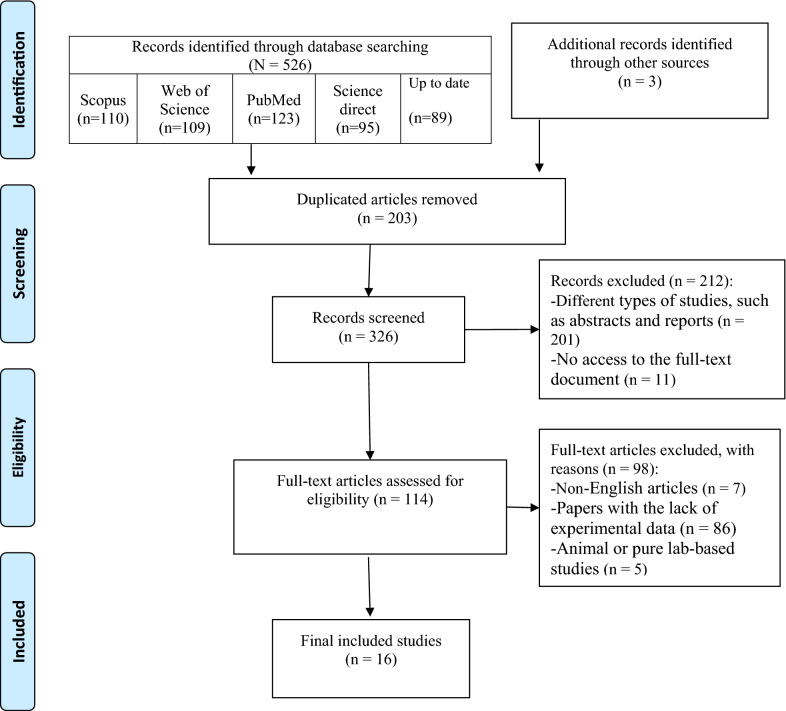
PRISMA 2020 flow diagram for systematic literature review

Our initial search of online databases retrieved 529 records. There were 203 duplicate records, resulting in the remainder of 326 records for the subsequent steps. About 114 articles entered the full-text screening step, and finally, 16 eligible records were selected for data extraction. The high quality of the included studies was validated by the results of the NOS quality assessment. Studies with a score of six or above were deemed to be of high quality. Three studies received a score of 9, eight received a score of 8, four received a score of 7, and one received a score of 6. No study had a score lower than six. The PRISMA Flow Diagram (Fig. 1) illustrates the details of our selection process.

The studies were divided based on their study population. Two studies were conducted among healthcare providers (1029 HIV healthcare providers in China) and one in HIV clinics (27 clinics in the USA). The remaining 13 studies were conducted among more than 21000 HIV-positive patients from 10 countries, including the USA (n = 4), Italy (n = 2), Argentina, Uganda, Pakistan, South Africa, Ethiopia, Indonesia, Belgium, and Brazil (n = 1 each). One of the studies [21], a cross-sectional online survey, had respondents from 26 countries but the majority of the study subjects were from Belgium and Brazil. In the majority of studies, the variables of interest (such as adherence to antiretroviral medications, increased anxiety, depression, and other mental health problems) were evaluated based on how often PLWH utilized care services and how frequently they skipped medications or doctor's appointments using questionnaires and patients' medical records.

### HIV-directed care challenges

In this review, 11 out of 13 studies conducted among people living with HIV (PLHIV) and one of two studies carried out among healthcare providers, reported a decrease (statistically significant or non-significant) in receiving or adhering to ART medications mostly due to the pandemic-related conditions [21–31].

#### Medication

The studies carried out among PLHIV reported alterations in ART uptake as follows: a rate of 33.7%,less-than-excellent and 1.7% under-average adherence to antiretrovirals medications [22], an increase of 7% (from 5 to 12%) in the rate of non-adherence to HIV medications [23], a 23.1% decrease in obtaining antiretroviral medication[24], a rate of 8.2% one-dose-ART-medication miss and 13.2% ART uptake avoidance [25], 5% to 26% increase in the chance of ART supply failure [26], a 36% temporary non-adherence due to inadequate ART supply [27], a 13.25% rate of running out of ART medication during pandemic [28], a 27.4% rate of missing visits for the antiretroviral stock refill [29], a 3% decrease in ART uptake [30], a 24.0% rate of missing at least one dose of HIV medications [31], and a 3.6% of ART refill failure [21]. In addition, one study conducted among providers reported a 67.25% rate of ART-application-service suspension or postponement due to the COVID-19 pandemic [32].

In our systematic review, almost all studies reported a decline in ART supply and uptake amid the pandemic [21–33]; however, in few studies, these changes had caused a statistically significant decrease in ART adherence. One study mentioned age ≥ 55, fear of COVID-19, transport disruption, reduced income and unaffordability traveling to healthcare facilities, limited access to face masks, and non-medical support as predicting factors for missing ART stock refill [29]. Another study reported healthcare accessibility disruption, clinical missed appointments, PTSD, and younger age as associated factors of missed doses of ART medications [31]. These predicting factors were mostly consistent with the main reasons described by the WHO including inadequate ART supply, the altered capacity of the health system that diverted healthcare workers toward COVID-19 management, transportation lockdown, and the shutdown of certain pharmaceutical companies [34]. Similarly, another included study in this review conducted among healthcare providers reported ART application service suspension or postponement due to COVID-19 restrictions or short supplies [32]. A similar study carried out in Northwest Ethiopia reported a substantial decline in ART services between 2019 and 2020 due to a full national lockdown [35]. Another similar study in China reported difficulties with refilling ART stocks due to lockdowns [36]. A similar study in China reported that 35.1% of PLHIV reported some sort of ART interruption, and 2.7% experienced ART shortage during the COVID-19 pandemic [37]. Also, many studies reported the new challenges ART-producing pharmaceuticals faced, amid the pandemic including problems with international shipping due to border restrictions, transportation delays, increased lead times, and increasing costs; which are some of the major reasons for global ART disruptions [38, 39]. One study carried out in Northwest Ethiopia noted social stigma or discrimination, missed HIV clinical visits, tuberculosis therapy, CD4 cell count < 500 cells/mm3, and being in the WHO-HIV clinical stage III at the time of ART initiation as predicting factors of low adherence to ART medications among their sample population. In a study conducted in Zimbabwe, researchers found that women on ART medications had problems accessing ART during the COVID-19 lockdown due to several factors including transportation difficulties, COVID-19 restrictions, police abuse, short medication supply, clinical check-up disruptions, involuntary ART non-adherence, and personal protective equipment (PPE) shortage [40]. In China, a survey reported that 32.64% of HIV-positive patients in the study did not possess enough ART to meet their actual needs [36]. One study that investigated the impacts of the COVID-19 pandemic on PLHIV, reported a considerable positive-screened rate of,respectively, 23.3% and 22.7% for major depressive disorders and anxiety disorders among their study population [41].

#### Appointments

Almost all studies reported challenges with clinical appointments and HIV follow-up sessions. Twelve studies reported that the pandemic has harmed clinical appointments among PLHIV [21, 23, 25–33, 42]; however, two studies reported an increase in the percentage of visits [24, 43]. Many patients had missed their HIV clinical visits, recovery support meetings, follow-up tests, and counseling services. The main causes of this general decline in clinical appointments were reported as follows: inadequate transport, police abuse, insufficient transportation funds to avoid exposure to COVID-19 [27], lockdowns [28], limited access to health services, reduced income, and inability to afford traveling to the health facility, being unable to get facemask, fear of COVID-19 [29], and fear of visiting hospitals [30]. Similarly, two studies conducted among healthcare providers reported disruptions in HIV services amid the pandemic. One study reported 22.0% complete; 15.4% moderate; and 21.9% minor disruption in HIV services [32], and another study reported 11.1% minimal; 56% partial; and 26% complete interruption among HIV clinics [42].

#### Healthcare

Healthcare provision for PLHIV is crucial to maintain viral suppression, lower HIV transmission, improve clinical outcomes, and expand lifespan [44]. While due to COVID-19 pandemic restrictions many PLHIVs are at great risk of missing clinical follow-ups and healthcare provisions, which eventually would lead to ART interruption. Many studies included in this review reported a form of disruption in HIV and general healthcare services as follows; HIV clinical visit misses [21, 24, 26–29, 31, 43], fear of visiting hospitals[30], and HIV clinic disruptions due to nationwide restrictions and healthcare diversion [32, 42]. A study carried out in Northwest Ethiopia reported a significant decrease in HIV services including HIV voluntary counseling and testing (VCT), and Provider-Initiated HIV Testing & Counseling (PITC) in 2020 compared to 2019 [35]. A similar study in Tigray, Northern Ethiopia reported a significant decline in HIV early diagnosis and detection, ART enrolment care, and general HIV care [45]. Another study carried out in China reported a significant decline in HIV testing rates in the first months of COVID-19 restrictions, a 37.0% lower rate of new HIV diagnoses compared to the expected rate, a 50.5% lower rate of CD4 cell count testing [46]. In addition, lower access to HIV clinical services reported by all these articles would result in higher HIV transmission, higher opportunistic infections, lower CD4 cell counts, and lower viral suppression [47]. Therefore, the healthcare provider and HIV services engagement with PLHIV is vital to maintain their clinical conditions and curb HIV transmission according to UNAIDS 90–90-90 policy [48].

Two studies reported an increase in telehealth utilization as a helpful method of avoiding COVID-19 exposure [21, 24]; while, one study reported difficulties in using telemedicine [22]. Three studies reported some forms of decrease or avoidance concerning the general healthcare during the pandemic [25, 28, 33]. Two studies conducted among healthcare providers reported suspension or postponement of services at HIV clinics, voluntary counseling, testing services, follow-up services, home visits, support groups, walk-in services, and the inability to a timely shift to a telehealth system [32, 42].

#### Viral suppression

Only two studies reported alteration in viral suppression during the pandemic and interestingly both of them reported an increase (not decrease) in viral suppression [26, 43]. However there does not seem to be sufficient data to conclude that there was no negative impact on the viral suppression. Detailed findings of all included studies and demographic information are presented in Table 2.

**Table 2:** Included studies’ summary of findings

Study info					Demographics						
ID	Author	Year	Country	Study type	Study population (N)	Age	%Male	Ethnicity	Education	Employment	Covid-19 infection
1	Ballivian et al. [22]	2020	Argentina	Quantitative Survey	1336 HIV +	45.83 ± 10.34	66.8	NA	NA	NA	NA
2	Giacomelli et al. [43]	2021	Italy	Retrospective study (before and after quasiexperimental design)	NA	NA	NA	NA	NA	NA	NA
3	Hochstatter et al. [23]	2021	USA	Quasi-experimental (time-series design)	60 HIV +	49 (Mean age)	75	White, Black/African American, Mixed, Hispanic/Latino	NA	Employed (39%)	NA
4	Quiros-Roldan et al. [24]	2020	Italy	Retrospective Observational Study	3875 HIV +	51.4 ± 13	72	Non-Italian	NA	NA	Hospitalized (48%)
5	Ridgway et al. [49]	2020	USA	NA	98 HIV +	NA	NA	African American	NA	NA	COVID + (14.5%), Hospitalized (75%)
6	Tamargo et al. [25]	2021	USA	Quantitative Survey	183 HIV +	56.5 ± 6.5	50.3	Non-Hispanic Black	NA	Unemployed (86.9%), Employed but on leave/had reduced hours (6.6%), Employed with no change (6.6%)	COVID + (7.6%), Hospitalized (0.6%)
7	Wagner et al. [26]	2021	Uganda	Observational Cohort Study	14,632 HIV +	38.7 ± 0.11	34	NA	NA	NA	NA
8	Ahmed et al. [27]	2022	Pakistan	Semi-structured interviews	25 HIV +	41	68	NA	No education (56%)	Unemployed (72%)	NA
9	Campbell et al. [28]	2021	South Africa	Cluster randomised control trial	83 HIV +	31	29	NA	Secondary (38.55%), Diploma (2.41%)	NA	NA
10	Chilot et al. [29]	2021	Ethiopia	Cross-Sectional Study	212 HIV +	48.6% in the age group 35–54 years	37.3	NA	No education (21.7%), Can read and write (14.2%), Primary education (27.8%), Secondary education (20.8%), Diploma and above ( 15.6%)	NA	NA
11	Karjadi et al. [30]	2021	Indonesia	Cross-Sectional Study	545 HIV +	66.5% in age group 36–55 years	72.8	NA	High-school graduate (49.5%)	NA	NA
12	Nguyen et al. [31]	2021	USA	Cross-sectional surveys	100 HIV +	64.2	96	NA	College education or higher (93.9%)	NA	NA
13	Rhodes et al. [33]	2020	South of USA	Qualitative study	15 HIV + cisgender men	28	100	Black/African American (50%), Latina (33%), White (27%)	NA	NA	NA
14	SieweFodjo et al. [21]	2021	26 countries, with a majority of respondents residing in Brazil (n = 83) and Belgium (n = 82)	Cross-sectional online survey	247 HIV +	44.5 ± 13.2	73.7	NA	Primary (7.7%), Secondary (29.6%), Undergraduate (30.4%), Post-graduate (32.4%)	NA	COVID + (15.3%)
15	Yang et al. [32]	2021	China	Cross-sectional Online Survey	1029 HIV healthcare providers	NA	NA	NA	NA	NA	NA
16	Qiao et al. [42]	2021	USA	Mixed-method study	27 HIV Clinics	NA	NA	NA	NA	NA	NA

### Non-HIV directed care challenges

Three studies reported an increase in depression, anxiety, and other mental disorders among PLWH [21, 28, 31] while two studies reported an interruption of therapeutic services to HIV-positive patients during the pandemic [22]. Alcohol and substance abuse were reported in one study as a disruption in substance abuse care services [21] and two studies reported increased use of recreational drugs. However, one study stated the opposite. Furthermore, HIV-positive patients encountered several problems concerning the COVID-19 care and treatment including stigma and discrimination associated with HIV [27], and difficulty in obtaining other medications [22] (Table 3).

**Table 3:** HIV and Non-HIV Directed Care Challenges

HIV directed care challenges						Non-HIV directed care challenges		
ID	Medication	Appointments	Health care	Viral Suppression	Other	Mental health care	Alcohol and substance abuse care	Other
1	Difficulty obtaining HIV medication (3.9%), Under average adherence to antiretrovirals (1.7%)	NA	Inability to access telehealth technology (34.7%)	NA	NA	Disruption in mental health services (11%)	Disruption in substance abuse care services (1.3%)	Difficulty obtaining other medication (9.1%)
2	No change in adherence to antiretrovirals	Increased interruption of virological follow-up in 2020 (12.9%) [half of them continued obtaining medication] / Decreased routine in-person appointments	NA	Progressive increase of virological suppression rate, a Decreasing trend of virological determinations ≥ 50 copies/mL during the pandemic	NA	NA	NA	NA
3	Decreased adherence to medication (from 5 to 12%)	Decreased attendance to recovery support meetings, Decrease in confidence to keep next HIV appointment	NA	NA	NA	NA	No difference in the proportion of people using alcohol (41%) or marijuana (32%), Increase utilization of illicit substances (including heroin, cocaine, sedatives, prescription opioids, or methamphetamine) (8%)	NA
4	23.1% decrease in obtaining medication during the pandemic. [higher in females and non-Italians]	Increased missing HIV clinic visits [higher in females]	Started using telemedicine (67.3%)	NA	Increase in the number of hospitalizations in HIV + patients	NA	NA	NA
5	Increased mail delivery for prescriptions and extended refills	visits during the pandemic: 2.0% attended in-person, 21.4% attended virtual visits, and 30.6% rescheduled for a later date. [rescheduled or canceled visits were associated with lower viral loads]/	NA	NA	The difficulty oflaboratory testing	Increased therapy session	NA	NA
6	No changes in antiretroviral adherence or health care utilization. [8% missed at least one dose and 13% avoided picking up ART due to the pandemic]	Missed an appointment in the past month (6.0%) [54.6% due to cancellations]	Avoided health care in the past month (20.2%)	NA	NA	NA	Reduced cocaine use and cigarette smoking	NA
7	No change (uncertain) in adherence to medication, Decreased obtaining medication and less refill during and post lockdown, No change in consistency of daily pill-taking timing, Missing a dose of ART (12.1%) [due to food insecurity during the lockdown]	Decreased clinic visits during lockdown [46% decreased compared to last year]	NA	No reduction with a slight increase in viral suppression (90%)	NA	NA	NA	NA
8	Temporarily non-adherent due to finishing off ART stock (36%) [presence of family improved the condition]	Difficulty visiting clinics [due to inadequate transport, police abuse, and insufficient transportation funds to avoid exposure to COVID-19]	NA	NA	Declined courier home delivery due to stigma	NA	NA	Covid care concerns regarding stigma and discrimination associated with HIV
9	No change in adherence, Run out of medication (13.25%)	Missed an appointment (15.66%)	Missed the community health worker's support (69.88%)	NA	NA	Depression (29%)	NA	Decrease in reporting violence (emotional, physical, sexual)
10	Missed visits for a refill (27.4%) [predicting factors: age ≥ 55, fear of COVID-19, transport disruption, reduced income for traveling to the health facility, and limited access to mask, sanitizer, and non-medical support]	Missed follow-up tests (26.4%), Missed counseling services (25.9%)	NA	NA	NA	NA	NA	NA
11	Stopped taking ART (3%)	Fear of visiting the hospital (48%)	NA	NA	NA	NA	NA	NA
12	Missed a dose of HIV medications (24.0%). [associated with disruption of access to health care, missed appointment, and PTSD] [younger respondents were more likely to report missed HIV medication doses]	Missed appointment (46.0%)	NA	NA	NA	Changes in sleep patterns (54.0%), Feeling anxious (56.0%), Frustrated (50.0%), Depressed (41%), Bored (43.0%)	NA	NA
13	No change in obtaining HIV medications, Difficulty adhering to medication regimens	Challenges with virtual appointments	Difficulty accessing medical care in the context of COVID-19	NA	NA	NA	NA	NA
14	Unable to refill their ART (3.6%)	Unable to meet their HIV physician face-to-face (55.8%)	NA	NA	NA	Anxiety and depression (27.9%), Anxiety and depression (19.8%), Need for psychosocial support (40.1%)	Increased recreational drug use (8.6)	NA
15	Providers reported ART application service was suspended or postponed because of COVID-19 (67.25%), Providers reported ART provision was suspended or postponed due to the short supply (0.49%)	NA	Providers reported HIV clinic service was suspended because of COVID-19 (5.05%), Reported voluntary counseling and testing service was suspended or postponed (53.94%), Reported outreach work was suspended or postponed (34.11%), and reported follow-up service was suspended or postponed (7.39%)	NA	NA	NA	NA	NA
16	NA	Most HIV clinics: Reduced their office hours, limited office visits, and limited face-to-face appointments, seven clinics were closed with only staff checking emails daily	HIV related services were interrupted at many clinics (scope and delivery), Two HIV clinics reported discontinued home visits and support groups, Several clinics suspended walk-in services, their Inability to timely shift to a telehealth system, and HIV clinics: were partially interrupted (56%) or completely closed (26%)	NA	Most of the clinics still provided core HIV services (e.g., medicine refilling, HIV testing), Suspended or canceled face-to-face counseling, and social support groups	NA	NA	NA

## Discussion

Management of chronic infections requires a steady process to obtain optimal outcomes. This process may engage all levels of healthcare-related management processes. Hence, disruption at any point will eventually affect this continuous process of managing chronic diseases like HIV. The COVID-19 pandemic has undoubtedly disturbed the global healthcare system to varying degrees. Countries that experienced the greatest disruption in the provision of healthcare services were mainly low or middle-income countries (LMICs). HIV infection management was therefore not spared from the impacts that the COVID-19 pandemic had on health services delivery. The detrimental impact of the COVID-19 pandemic on the treatment of PLHIV has been documented in several aspects of disease management. From the analysis of this study, the COVID-19 pandemic created several challenges concerning HIV- and non-HIV-directed care as well as healthcare delivery. Our findings show that there has been a decline in adherence to ART among PLHIV, although no impact on viral suppression was observed. Depression, anxiety, substance abuse, stigma, and discrimination were observed as the prominent non-HIV-directed care challenges posed by the current pandemic. With regards to the healthcare provision and delivery, in-person visits and clinical follow-up services appeared to be greatly impaired by the pandemic, although the use of telemedicine helped to alleviate the situation to a great extent.

### HIV-directed care challenges

Since the beginning of the COVID-19 pandemic, research has shown that patients with underlying health conditions including diabetes mellitus, cardiovascular diseases, hypertension, and cancers are more susceptible to experiencing moderate-severe COVID-19 infection, poor prognosis, and higher mortality rates compared to the general population [50]. However, the results on the risk of COVID-19 infection, its related morbidities, and mortality among HIV-positive patients are controversial. Some included studies have reported that there is no significantly higher risk of developing severe COVID-19 among PLHIV compared to the general population [51–53] or even advanced-stage HIV patients with lower CD4 counts that are severely immunocompromised may show less severe COVID‐19 symptoms [54]. On the other hand, some studies have shown that PLHIV may be at a greater risk of COVID-19 complications [55]. In addition, a meta-analysis reported a 2- to threefold increase in mortality rates from COVID-19 among PLHIV compared to the general population [56].

### Antiretroviral therapy (ART) disruption

COVID-19 pandemic has caused a substantial negative impact on HIV services and ART distribution-which are to prevent HIV progression and prolong the PLHIV lifespan- around the globe. According to WHO the percentage of PLHIV taking ART medications was 73% in 2020 [57]. In June 2020, WHO reported that there was a global disruption in HIV services, and at the same time a widespread disruption in ART services in 36 countries had been documented by WHO, affecting 11.5 million people in these countries who were using ART (45% of total PLHIV), and also 24 countries suffered from inadequate ART supply, impacting 8.3 million people of the population receiving ART in these countries (33% of total PLHIV on ART) [34, 58]. According to a model created by WHO and UNAIDS, a 6-month disruption of ART drug supplies among half of PLHIV would lead to a 1.63-fold increase in HIV-related mortalities worldwide [59]. This emphasizes the importance of ART drug distribution, stock refill, and clinical follow-up of HIV services continuation.

In our systematic review, almost all studies reported a decline in ART supply and uptake amid the pandemic [21–33]; however, in few studies, these changes had caused a statistically significant decrease in ART adherence. One study mentioned age ≥ 55, fear of COVID-19, transport disruption, reduced income and unaffordability traveling to healthcare facilities, limited access to face masks, and non-medical support as predicting factors for missing ART stock refill [29]. Another study reported healthcare accessibility disruption, clinical missed appointments, PTSD, and younger age as associated factors of missed doses of ART medications [31]. These predicting factors were mostly consistent with the main reasons described by the WHO including inadequate ART supply, the altered capacity of the health system that diverted healthcare workers toward COVID-19 management, transportation lockdown, and the shutdown of certain pharmaceutical companies [34]. Similarly, another included study in this review conducted among healthcare providers reported ART application service suspension or postponement due to COVID-19 restrictions or short supplies [32]. A similar study carried out in Northwest Ethiopia reported a substantial decline in ART services between 2019 and 2020 due to a full national lockdown [35]. Another similar study in China reported difficulties with refilling ART stocks due to lockdowns [36]. A similar study in China reported that 35.1% of PLHIV reported some sort of ART interruption, and 2.7% experienced ART shortage during the COVID-19 pandemic [37]. Also, many studies reported the new challenges ART-producing pharmaceuticals faced, amid the pandemic including problems with international shipping due to border restrictions, transportation delays, increased lead times, and increasing costs; which are some of the major reasons for global ART disruptions [38, 39]. One study carried out in Northwest Ethiopia noted social stigma or discrimination, missed HIV clinical visits, tuberculosis therapy, CD4 cell count < 500 cells/mm3, and being in the WHO-HIV clinical stage III at the time of ART initiation as predicting factors of low adherence to ART medications among their sample population. In a study conducted in Zimbabwe, researchers found that women on ART medications had problems accessing ART during the COVID-19 lockdown due to several factors including transportation difficulties, COVID-19 restrictions, police abuse, short medication supply, clinical check-up disruptions, involuntary ART non-adherence, and personal protective equipment (PPE) shortage [40]. In China, a survey reported that 32.64% of HIV-positive patients in the study did not possess enough ART to meet their actual needs [36]. One study that investigated the impacts of the COVID-19 pandemic on PLHIV, reported a considerable positive-screened rate of,respectively, 23.3% and 22.7% for major depressive disorders and anxiety disorders among their study population [60].

Thus, it is obvious that a shortage of ART medications is of great danger for PLHIV in terms of physical, psychological, and social wellbeing. To address this issue many countries and HIV services worldwide took measures such as sending medications via mail delivery, extended ART stock refills [61], incorporating telemedicine for healthcare provision continuation and decreasing the risk of exposure [62], broadening access to ART multi-month dispensing (MMD) policy, concentrating on community-based case-finding, and immediate, initiating ART among newly-diagnosed cases [63], launching telephone consultations, expanding ART refill sites [60], and HIV-positive patients task-shifting for early ART refill collection [64]. Hopefully, these measures assisted HIV services substantially to mitigate COVID-19-related ART disruptions and eventually caused an early return to normal ART adherence. According to the latest WHO reports, in November 2020, only 9 countries had reported disruptions in ART services, and only 12 countries had critically low ART stocks, this was mostly a result of putting MMD policies into action during the COVID-19 pandemic by 90% of countries globally [34].

### HIV and general healthcare disruption

Healthcare provision for PLHIV is crucial to maintain viral suppression, lower HIV transmission, improve clinical outcomes, and expand lifespan [44]. While due to COVID-19 pandemic restrictions many PLHIVs are at great risk of missing clinical follow-ups and healthcare provisions, which eventually would lead to ART interruption. Many studies included in this review reported a form of disruption in HIV and general healthcare services as follows; HIV clinical visit misses [21, 24, 26–29, 31, 43], fear of visiting hospitals[30], and HIV clinic disruptions due to nationwide restrictions and healthcare diversion[32, 42]. A study carried out in Northwest Ethiopia reported a significant decrease in HIV services including HIV Voluntary counseling and testing (VCT), and Provider-Initiated HIV Testing & Counseling (PITC) in 2020 compared to 2019 [35]. A similar study in Tigray, Northern Ethiopia reported a significant decline in HIV early diagnosis and detection, ART enrolment care, and general HIV care [45]. Another study carried out in China reported a significant decline in HIV testing rates in the first months of COVID-19 restrictions, a 37.0% lower rate of new HIV diagnoses compared to the expected rate, a 50.5% lower rate of CD4 cell count testing [46]. In addition, lower access to HIV clinical services reported by all these articles would result in higher HIV transmission, higher opportunistic infections, lower CD4 cell counts, and lower viral suppression [47]. Therefore, the healthcare provider and HIV services engagement with PLHIV is vital to maintain their clinical conditions and curb HIV transmission according to UNAIDS 90-90-90 policy [48].

One of the best methods that gained huge popularity amid the COVID-19 pandemic was telemedicine [65], which is a method of delivering healthcare via telephone, internet, or other methods of telecommunication. Two studies included in our review reported that PLHIV benefited from telemedicine during the pandemic [21, 24], while one study reported some difficulties utilizing telehealth facilities for HIV care [22]. Telemedicine has been harnessed during the COVID-19 pandemic for a variety of uses including out-patient consultation, inpatient care, emergency triage, patient hospitalization, interhospital consult, and patient education via phone and video calls [66], and has assisted many countries with controlling COVID-19 pandemic and maintain healthcare for vulnerable populations [67]. However, telehealth accessibility generally relies on age, level of education, and economic status, as most high-income countries utilized this method of healthcare provision. Thus, low-income countries must improve their infrastructure to increase telemedicine availability for their population and transform health care globally [68, 69].

### Viral suppression

Finally, only two studies included in this review investigated the viral suppression among PLHIV and surprisingly they reported progressive increasing rates [26, 43]. However, the data was inadequate to determine that there was no negative impact on viral suppression. Similarly in a time-series study conducted at San Francisco General Hospital viral suppression did not alter [70]. Another study from Italy reported that positive-viremic patients during the COVID-19 period were lower in 2020 compared to 2019 [71]. All these studies noted that the measures taken by countries during the COVID-19 pandemic to mitigate the negative impacts on healthcare delivery continuation were useful.

### Non-HIV directed care challenges

People living with HIV endure a lot of challenges; not only related to medications, but also several other issues concerning social life, psychological status, and mental health. Social life challenges faced by PLHIV may be perceived as discrimination and stigmatization [72, 73]. Without the COVID-19 pandemic, social discrimination and stigmatization are prominent issues faced by PLHIV [74, 75]. The evidence indicated that these challenges could also be experienced by PLHIV even during the pandemic [22, 27]. Interestingly, studies have reported that PLHIVs were obliged to change their health center and reported losing their jobs and even being denied health services during the pandemic [29, 30]. Hence, there is still the need to probe further into structural causes and possible interventions in dealing with the stigma associated with HIV status.

Mental health services were not left out in the challenges posed by the pandemic on PLHIV. The COVID-19 pandemic negatively affected the provision of mental health services in this vulnerable population [22, 28]. As a result, current evidence has shown that the rate of depression, anxiety, sleep disturbance, and boredom have increased in PLHIV from 20 to 55% [21, 28, 31]. Although these mental health issues existed before the pandemic in these patients, a systematic review hypothesizes that the COVID-19 pandemic has exacerbated the condition [72], hence, this issue may require prompt attention and immediate actions. Furthermore, analysis of the current evidence indicates that there is a link between reduced mental health services for PLHIV and other social challenges such as the disruption in illicit substance-abuse-care services [22], an increase in the utilization of illicit substances (including heroin, cocaine, sedatives, prescription opioids, or methamphetamine) from 10 to 18% [23] and increased recreational drug use [21, 22].However, very few studies propose that there is no significant change in the use of illicit drugs among PLHIV during the pandemic [23, 25]. This negative impact of the pandemic may not only make PLHIV more liable to infection with COVID-19 but will also go a long way in disrupting the treatment and management of HIV infection [76–80].

Other non-healthcare challenges reported from our analysis include consumption of alcohol, food insecurity [21, 25, 26], increased abuse during quarantine [22, 28], and financial difficulties [28–31]. Additionally, various studies have reported that alcohol appears to increase the risk of COVID-19 infection and complicate the disease progression. Moreover, alcohol use could exacerbate the problem of the pandemic, particularly among vulnerable groups such as adolescents, the elderly, cancer patients, and chronic disease patients including PLHIV. It has been observed that the services for patients suffering from alcoholism have been impacted by the pandemic [81–83]. The observed trends during the pandemic further disrupted the steady care that is needed for PLHIV.

## Limitations and recommendations

To improve the provision of services to people living with HIV during and after the pandemic, targeted solutions must be developed. One of the suggested methods is remote medical visits to maintain PLHIVs' constant access to their providers and medical services and consultation; those otherwise they are unable to receive due to stigma, discrimination, or far distance from medical centers, especially in countries with higher HIV rates.

Given that the disruption in the supply of ART drugs during the pandemic has led to an increase in mortality, it seems that special attention to the timely supply of ART, the availability of sufficient additional drugs in stock, and clinical follow-up of HIV services continuation are necessary. To maintain the treatment process of PLHIV, their access to ART should be a priority, and treatment guidelines should explicitly emphasize the importance of easy access to ART. There are shreds of evidence about sufficient psychological treatments for common psychological problems of PLHIV, including anxiety, depression, alcohol, and substance use disorders. During COVID-19, these interventions should be delivered using mobile technology and social media. In addition, in LMICs, due to their impaired access to mobile phones and technology, culturally acceptable and feasible psychological interventions should be developed and implemented as a crucial part of regular HIV care.

## Conclusion

In summary, interruption in face-to-face visits and follow-ups, lack of treatment adherence and subsequent increase in mortality from COVID-19 pandemic complications in PLHIV has led to growing concerns. Other challenges of the current pandemic in PLHIV were psychological disorders such as anxiety and depression, an increase in substance use, and a rise in experienced stigma and discrimination. The experiences of countries that have used telemedicine for HIV care and management during the pandemic, should be shared globally and used as a potential measure to improve service delivery in other countries, especially LMICs countries with weak infrastructures.

## Data Availability

The authors stated that all information provided in this article could be shared.

## References

[1] Dadras O, Afsahi AM, Pashaei Z, Mojdeganlou H, Karimi A, Habibi P (2022). The relationship between COVID-19 viral load and disease severity: a systematic review. Immun Inflam Disease..

[2] Mehraeen E, Salehi MA, Behnezhad F, Moghaddam HR, SeyedAlinaghi S (2021). Transmission modes of COVID-19: a systematic review. Infect Disord Drug Targets.

[3] WHO. Organization WH. Coronavirus Disease (COVID-19) - events as they happen. world health organization 2020 https://www.who.int/emergencies/diseases/novel-coronavirus-2019/events-as-they-happen.

[4] Mehraeen E, Oliaei S, SeyedAlinaghi S, Karimi A, Mirzapour P, Afsahi AM (2022). COVID-19 in pediatrics: a systematic review of current knowledge and practice. Infect Disord Drug Targets.

[5] SeyedAlinaghi S, Karimi A, Mojdeganlou H, Alilou S, Mirghaderi SP, Noori T (2022). Impact of COVID-19 pandemic on routine vaccination coverage of children and adolescents: a systematic review. Health Sci Rep.

[6] Mukwenha S, Dzinamarira T, Mugurungi O, Musuka G (2020). Maintaining robust HIV and tuberculosis services in the COVID-19 era: a public health dilemma in Zimbabwe. Int J Infect Dis.

[7] Moitra E, Tao J, Olsen J, Shearer RD, Wood BR, Busch AM (2022). Impact of the COVID-19 pandemic on HIV testing rates across four geographically diverse urban centres in the United States: an observational study. Lancet Reg Health Am.

[8] Jewell BL, Smith JA, Hallett TB (2020). Understanding the impact of interruptions to HIV services during the COVID-19 pandemic: a modelling study. EClinicalMedicine.

[9] Medina N, Alastruey-Izquierdo A, Bonilla O, Ortíz B, Gamboa O, Salazar LR (2021). Impact of the COVID-19 pandemic on HIV care in Guatemala. Int J Infect Dis.

[10] Dadras O, Alinaghi SAS, Karimi A, MohsseniPour M, Barzegary A, Vahedi F (2021). Effects of COVID-19 prevention procedures on other common infections: a systematic review. Eur J Med Res.

[11] Adugna A, Azanaw J, Melaku MS (2021). The effect of COVID-19 on routine HIV care services from health facilities in Northwest Ethiopia. HIV AIDS.

[12] Mehraeen E, Safdari R, Seyedalinaghi SA, Mohammadzadeh N, Arji G (2018). Identifying and validating requirements of a mobile-based self-management system for people living with HIV. Studies in health Technol Inform.

[13] O’Brien KK, Bayoumi AM, Chan Carusone S, Davis AM, Aubry R, Avery L (2021). Disability and self-care living strategies among adults living with HIV during the COVID-19 pandemic. AIDS Res Ther.

[14] Siewe Fodjo JN, de Moura Faria, Villela E, Van Hees S, Vanholder P, Reyntiens P, Colebunders R (2021). Follow-up survey of the impact of COVID-19 on people living with HIV during the second semester of the pandemic. Int J Environ Res Public Health.

[15] Mehraeen E, Safdari R, Mohammadzadeh N, Seyedalinaghi SA, Forootan S, Mohraz M (2018). Mobile-based applications and functionalities for self-management of people living with HIV. Stud Health Technol and Inform.

[16] Kanwugu ON, Adadi P (2021). HIV/SARS-CoV-2 coinfection: a global perspective. J Med Virol.

[17] Musuka G, Dzinamarira T, Madziva R, Herrera H, El Sadr W (2022). Protecting HIV service delivery for key populations in southern Africa in the context of the COVID-19 pandemic. IJID Regions.

[18] Mehraeen E, Safdari R, SeyedAlinaghi S, Noori T, Kahouei M, Soltani-Kermanshahi M (2020). A mobile-based self-management application—usability evaluation from the perspective of HIV-positive people. Health Policy Technol.

[19] SeyedAlinaghi S, Karimi A, MohsseniPour M, Barzegary A, Mirghaderi SP, Fakhfouri A (2021). The clinical outcomes of COVID-19 in HIV-positive patients: a systematic review of current evidence. Immunity, Inflam Disease.

[20] Dorward J, Khubone T, Gate K, Ngobese H, Sookrajh Y, Mkhize S (2021). The impact of the COVID-19 lockdown on HIV care in 65 South African primary care clinics: an interrupted time series analysis. The Lancet HIV.

[21] Siewe Fodjo JN, de Moura Faria, Villela E, Van Hees S, Vanholder P, Reyntiens P, Colebunders R (2021). Follow-up survey of the impact of COVID-19 on people living with hiv during the second semester of the pandemic. Int J Environ Res Public Health.

[22] Ballivian J, Alcaide ML, Cecchini D, Jones DL, Abbamonte JM, Cassetti I (2020). Impact of COVID-19-related stress and lockdown on mental health among people living with HIV in Argentina. J Acquir Immune Defic Syndr.

[23] Hochstatter KR, Akhtar WZ, Dietz S, Pe-Romashko K, Gustafson DH, Shah DV (2021). Potential Influences of the COVID-19 pandemic on drug use and HIV care among people living with HIV and substance use disorders: experience from a pilot mhealth intervention. AIDS Behav.

[24] quiros roldan E, Magro P, Carriero C, Chiesa A, Hamad I, Tratta E, et al. Consequences of the COVID-19 pandemic on the continuum of the care in a cohort of people living with HIV followed in a single center of Northern Italy 2020. AIDS Res Ther. 2020;17(1):59. 10.1186/s12981-020-00314-y PMC7533114 33012282

[25] Tamargo JA, Martin HR, Diaz-Martinez J, Trepka MJ, Delgado-Enciso I, Johnson A (2021). COVID-19 testing and the impact of the pandemic on the miami adult studies on hiv cohort. J Acquir Immune Defic Syndr.

[26] Wagner Z, Mukasa B, Nakakande J, Stecher C, Saya U, Linnemayr S (2021). Impact of the COVID-19 pandemic on use of HIV care, antiretroviral therapy adherence, and viral suppression: an observational cohort study from Uganda. J Acquir Immune Defic Syndr.

[27] Ahmed A, Dujaili JA, Jabeen M, Umair MM, Chuah L-H, Hashmi FK (2022). Barriers and enablers for adherence to antiretroviral therapy among people living with HIV/AIDS in the Era of COVID-19. Qualit Study Pakistan.

[28] Campbell LS, Masquillier C, Knight L, Delport A, Sematlane N, Dube LT (2022). Stay-at-home: the impact of the COVID-19 lockdown on household functioning and ART adherence for people living with HIV in three sub-districts of cape town South Africa. AIDS Behav.

[29] Chilot D, Woldeamanuel Y, Manyazewal T (2021). COVID-19 burden on HIV patients attending antiretroviral therapy in addis Ababa, ethiopia a multicenter cross-sectional study. Res Square.

[30] Karjadi TH, Maria S, Yunihastuti E, Widhani A, Kurniati N, Imran D (2021). Knowledge, attitude, behavior, and socioeconomic conditions of people living with HIV in Indonesia during the COVID-19 pandemic: a cross-sectional study. HIV AIDS.

[31] Nguyen AL, Davtyan M, Taylor J, Christensen C, Plankey M, Karpiak S (2021). Living with HIV during the COVID-19 pandemic: impacts for older adults in palm Springs, California. AIDS Edu Prev Off Pub Int Soc AIDS Edu.

[32] Yang X, Zeng C, Tam CC, Qiao S, Li X, Shen Z (2022). HIV service interruptions during the COVID-19 Pandemic in China: the role of COVID-19 challenges and institutional response from healthcare professional’s perspective. AIDS Behav.

[33] Rhodes SD, Mann-Jackson L, Alonzo J, Garcia M, Tanner AE, Smart BD (2021). A rapid qualitative assessment of the impact of the covid-19 pandemic on a racially/ethnically diverse sample of Gay, bisexual, and other men who have sex with men living with HIV in the US South. AIDS Behav.

[34] World Health Organization. HIV & COVID-19 2020. https://www.who.int/teams/global-hiv-hepatitis-and-stis-programmes/covid-19

[35] Adugna A, Azanaw J, Sharew MM (2021). The effect of COVID-19 on routine HIV care services from health facilities in Northwest Ethiopia. HIV AIDS.

[36] Guo W, Weng HL, Bai H, Liu J, Wei XN, Zhou K (2020). Quick community survey on the impact of COVID-19 outbreak for the healthcare of people living with HIV. Zhonghua Liu Xing Bing Xue Za Zhi.

[37] Sun Y, Li H, Luo G, Meng X, Guo W, Fitzpatrick T (2020). Antiretroviral treatment interruption among people living with HIV during COVID-19 outbreak in China: a nationwide cross-sectional study. J Int AIDS Soc.

[38] Rewari B, Mangadan-Konath N, Sharma M (2020). Impact of COVID-19 on the global supply chain of antiretroviral drugs: a rapid survey of Indian manufacturers. WHO South-East Asia J Public Health.

[39] Times. P. Drug shortage concerns are top of mind amid COVID-19 outbreak https://www.pharmacytimes.com/news/drug-shortage-concerns-are-top-of-mind-amid-covid-19-outbreak Accessed 23 March 2020

[40] Nyashanu M, Chireshe R, Mushawa F, Ekpenyong MS (2021). Exploring the challenges of women taking antiretroviral treatment during the COVID-19 pandemic lockdown in peri-urban Harare, Zimbabwe. Int J Gynecol Obstet.

[41] Fodjo JNS, de Moura Villela EF, Van Hees S, Dos Santos TT, Vanholder P, Reyntiens P (2020). Impact of the COVID-19 pandemic on the medical follow-up and psychosocial well-being of people living with HIV: a cross-sectional survey. J Acq Immune Defic Syn.

[42] Qiao S, Li Z, Weissman S, Li X, Olatosi B, Davis C (2021). Disparity in HIV service interruption in the outbreak of COVID-19 in South Carolina. AIDS Behav.

[43] Giacomelli A, Bonazzetti C, Conti F, Pezzati L, Oreni L, Micheli V (2021). Brief report: impact of the COVID-19 pandemic on virological suppression in people living with hiv attending a large Italian HIV clinic. J Acquir Immune Defic Syndr.

[44] Mugavero MJ, Amico KR, Horn T, Thompson MA (2013). The state of engagement in HIV care in the United States: from cascade to continuum to control. Clin Infect Dis.

[45] Desta AA, Woldearegay TW, Gebremeskel E, Alemayehu M, Getachew T, Gebregzabiher G (2021). Impacts of COVID-19 on essential health services in Tigray, Northern Ethiopia: a pre-post study. PLoS ONE.

[46] Shi L, Tang W, Hu H, Qiu T, Marley G, Liu X (2021). The impact of COVID-19 pandemic on HIV care continuum in Jiangsu, China. BMC Infect Dis.

[47] Jones LE, Perelson AS (2007). Transient viremia, plasma viral load, and reservoir replenishment in HIV-infected patients on antiretroviral therapy. J Acquir Immune Defic Syndr.

[48] Joint United Nations Programme on HIV/AIDS. 90-90-90: treatment for all https://www.unaids.org/en/resources/909090 Accessed 1 Jan 2017.

[49] Ridgway JP, Schmitt J, Friedman E, Taylor M, Devlin S, McNulty M (2020). HIV care continuum and COVID-19 outcomes among people living with HIV during the COVID-19 pandemic, Chicago. IL AIDS Behav.

[50] World Health Organization. COVID-19: vulnerable and high risk groups https://www.who.int/westernpacific/emergencies/covid-19/information/high-risk-groups Accessed 25 March 2020.

[51] Sigel K, Swartz T, Golden E, Paranjpe I, Somani S, Richter F (2020). Coronavirus 2019 and people living with human immunodeficiency virus: outcomes for hospitalized patients in New York City. Clin Infect Dis.

[52] Tesoriero JM, Swain CE, Pierce JL, Zamboni L, Wu M, Holtgrave DR (2021). COVID-19 outcomes among persons living with or without diagnosed HIV infection in New York State. JAMA Netw Open.

[53] Shi P, Ren G, Yang J, Li Z, Deng S, Li M (2020). Clinical characteristics of imported and second-generation coronavirus disease 2019 (COVID-19) cases in Shaanxi outside Wuhan, China: a multicentre retrospective study. Epidemiol Infect.

[54] SeyedAlinaghi S, Karimi A, MohsseniPour M, Barzegary A, Mirghaderi SP, Fakhfouri A (2021). The clinical outcomes of COVID-19 in HIV-positive patients: a systematic review of current evidence. Immun Inflamm Dis.

[55] So-Armah K, Benjamin LA, Bloomfield GS, Feinstein MJ, Hsue P, Njuguna B (2020). HIV and cardiovascular disease. Lancet HIV.

[56] Oyelade T, Alqahtani JS, Hjazi AM, Li A, Kamila A, Raya RP (2022). Global and regional prevalence and outcomes of COVID-19 in people living with HIV: a systematic review and meta-analysis. Trop Med Infect Dis.

[57] World Health Organization. Global HIV programme https://www.who.int/teams/global-hiv-hepatitis-and-stis-programmes/hiv/strategic-information/hiv-data-and-statistics Accessed 20 July 2021

[58] Shiau S, Krause KD, Valera P, Swaminathan S, Halkitis PN (2020). The Burden of COVID-19 in people living with HIV: a syndemic perspective. AIDS Behav.

[59] Gatechompol S, Avihingsanon A, Putcharoen O, Ruxrungtham K, Kuritzkes DR (2021). COVID-19 and HIV infection co-pandemics and their impact: a review of the literature. AIDS Res Ther.

[60] Siewe Fodjo JN, Villela EFdM, Van Hees S, dos Santos TT, Vanholder P, Reyntiens P (2020). Impact of the COVID-19 pandemic on the medical follow-up and psychosocial well-being of people living with HIV: a cross-sectional survey. J Acqu Imm Deficiency Syn.

[61] Ridgway JP, Schmitt J, Friedman E, Taylor M, Devlin S, McNulty M (2020). HIV Care Continuum and COVID-19 outcomes among people living with HIV During the COVID-19 pandemic, Chicago. IL AIDS and Behavior.

[62] Rockwell KL, Gilroy AS (2020). Incorporating telemedicine as part of COVID-19 outbreak response systems. Am J Manag Care.

[63] Boyd AT, Jahun I, Dirlikov E, Greby S, Odafe S, Abdulkadir A (2021). Expanding access to HIV services during the COVID-19 pandemic—Nigeria, 2020. AIDS Res Ther.

[64] Pry JM, Sikombe K, Mody A, Iyer S, Mutale J, Vlahakis N (2022). Mitigating the effects of COVID-19 on HIV treatment and care in Lusaka, Zambia: a before–after cohort study using mixed effects regression. BMJ Glob Health.

[65] Lukas H, Xu C, Yu Y, Gao W (2020). Emerging telemedicine tools for remote COVID-19 diagnosis, monitoring, and management. ACS Nano.

[66] Hincapié MA, Gallego JC, Gempeler A, Piñeros JA, Nasner D, Escobar MF (2020). Implementation and usefulness of telemedicine during the COVID-19 Pandemic: a scoping review. J Prim Care Commun Health.

[67] Marhefka S, Lockhart E, Turner D (2020). Achieve research continuity during social distancing by rapidly implementing individual and group videoconferencing with participants: key considerations, best practices, and protocols. AIDS Behav.

[68] Ramirez AV, Ojeaga M, Espinoza V, Hensler B, Honrubia V (2020). Telemedicine in minority and socioeconomically disadvantaged communities amidst COVID-19 pandemic. Otolaryngol Head Neck Surg.

[69] Doraiswamy S, Abraham A, Mamtani R, Cheema S (2020). Use of telehealth during the COVID-19 pandemic: scoping review. J Med Int Res.

[70] Hickey MD, Imbert E, Glidden DV, Del Rosario JB, Chong M, Clemenzi-Allen A (2021). Viral suppression during COVID-19 among people with HIV experiencing homelessness in a low-barrier clinic-based program. AIDS.

[71] Izzo I, Carriero C, Gardini G, Fumarola B, Chiari E, Castelli F (2021). Impact of COVID-19 pandemic on HIV viremia: a single-center cohort study in northern Italy. AIDS Res Ther.

[72] MacLean JR, Wetherall K (2021). The association between hiv-stigma and depressive symptoms among people living with HIV/AIDS: a systematic review of studies conducted in South Africa. J Affect Disord.

[73] Yuvaraj A, Mahendra VS, Chakrapani V, Yunihastuti E, Santella AJ, Ranauta A (2020). HIV and stigma in the healthcare setting. Oral Dis.

[74] Chambers LA, Rueda S, Baker DN, Wilson MG, Deutsch R, Raeifar E (2015). Stigma, HIV and health: a qualitative synthesis. BMC Public Health.

[75] Jackson-Best F, Edwards N (2018). Stigma and intersectionality: a systematic review of systematic reviews across HIV/AIDS, mental illness, and physical disability. BMC Public Health.

[76] Dubey MJ, Ghosh R, Chatterjee S, Biswas P, Chatterjee S, Dubey S (2020). COVID-19 and addiction. Diabetes Metab Syndr.

[77] Khalsa JH, Bunt G, Maggirwar SB, Kottilil S (2021). COVID-19 and cannabidiol (CBD). J Addict Med.

[78] Melamed OC, Hauck TS, Buckley L, Selby P, Mulsant BH (2020). COVID-19 and persons with substance use disorders: Inequities and mitigation strategies. Subst Abus.

[79] Volkow ND (2020). Collision of the COVID-19 and addiction epidemics. Ann Intern Med.

[80] Wang QQ, Kaelber DC, Xu R, Volkow ND (2021). COVID-19 risk and outcomes in patients with substance use disorders: analyses from electronic health records in the United States. Mol Psychiatry.

[81] Jones EAK, Mitra AK, Bhuiyan AR (2021). Impact of COVID-19 on mental health in adolescents: a systematic review. Int J Environ Res Public Health.

[82] Murthy P, Narasimha VL (2021). Effects of the COVID-19 pandemic and lockdown on alcohol use disorders and complications. Curr Opin Psychiatry.

[83] Ramalho R (2020). Alcohol consumption and alcohol-related problems during the COVID-19 pandemic: a narrative review. Australas Psychiatry.

